# Synergistic PdMoCu Trimetallic Metallene-Enhanced Electrochemiluminescence Biosensor for Ultrasensitive Detection of Microcystin-LR

**DOI:** 10.3390/bios16050264

**Published:** 2026-05-02

**Authors:** Xiaochen Yang, Linsheng Wang, Jing Tu, Yanlei Li, Lun Yang, Zhongfeng Gao

**Affiliations:** 1 Key Laboratory of Interfacial Reaction & Sensing Analysis in Universities of Shandong, School of Chemistry and Chemical Engineering, University of Jinan, Jinan 250022, China; 202321100270@stu.ujn.edu.cn (X.Y.); 202330222028@stu.ujn.edu.cn (L.W.); 202430222188@stu.ujn.edu.cn (J.T.); liyl@stu.ujn.edu.cn (Y.L.); 2 Faculty of Computer Science, University of Vienna, 1010 Vienna, Austria

**Keywords:** electrochemistry, functional nanomaterials, biosensors, aptamer

## Abstract

The development of highly sensitive and reliable strategies for microcystin-LR (MC-LR) monitoring remains critical for environmental safety and public health protection. Herein, we report a metallene-enabled electrochemiluminescence (ECL) biosensing platform based on ultrathin PdMoCu trimetallic metallenes for femtogram-level MC-LR detection. The two-dimensional PdMoCu metallenes provide abundant active sites and accelerated interfacial charge-transfer kinetics through synergistic electronic modulation among Pd, Mo, and Cu atoms, significantly enhancing the Ru(bpy)_3_^2+^/TPrA ECL efficiency. By integrating a programmable H1–aptamer duplex interface, electrostatic enrichment of Ru(bpy)_3_^2+^ was achieved, enabling target-responsive luminophore release via aptamer-triggered structural switching. This cooperative amplification mechanism, combining catalytic acceleration and DNA-mediated signal modulation, results in a sensitive signal-off detection mode. Under optimized conditions, the biosensor exhibited a wide linear response from 0.1 pg mL^−1^ to 50 ng mL^−1^ with a detection limit as low as 37 fg mL^−1^. The platform demonstrated excellent selectivity against structural analogues, high reproducibility, and satisfactory recovery (99.3–102.0%) in real tap water samples. This work not only highlights the catalytic potential of trimetallic metallenes in ECL systems but also establishes a generalizable interfacial engineering strategy for ultrasensitive detection of trace environmental contaminants.

## 1. Introduction

Microcystins are a group of potent toxins that are primarily produced by certain species of cyanobacteria, commonly referred to as blue-green algae [1,2]. These algae thrive in nutrient-rich environments, often forming harmful algal blooms (HABs) in water bodies, including lakes, rivers, and reservoirs. These blooms release microcystins into the water, which are incredibly stable and resistant to environmental changes. As a result, they persist in aquatic ecosystems, posing a significant health threat to both humans and animals [3,4]. Microcystins can be introduced into the human body through various routes, including ingestion of contaminated water or seafood, inhalation of aerosols during recreational activities, or direct skin contact with polluted water [5]. The toxins are known to cause liver damage, gastrointestinal issues, and even more severe health complications such as cancer and neurotoxicity [6,7]. Among the different types of microcystins, microcystin-LR (MC-LR) is considered the most toxic due to its strong affinity for the protein phosphatase enzymes in human cells, which disrupt cellular signaling pathways and cause irreversible damage [8]. Due to its high toxicity and persistence in aquatic ecosystems, the detection of MC-LR is a matter of paramount importance for safeguarding public health, promoting environmental protection, and advancing scientific knowledge [9,10,11]. Early detection of MC-LR contamination in water bodies is crucial as it allows for swift mitigation measures to prevent exposure, ensuring that drinking water, recreational water bodies, and food sources are safe for consumption and use [12,13,14]. Given the potential for microcystin contamination to cause widespread harm, there is an urgent need for reliable, rapid, and accurate methods to detect MC-LR in environmental and biological samples.

In recent years, considerable efforts have been dedicated to the development of innovative detection technologies aimed at the rapid and accurate identification of MC-LR in water samples. Traditional methods such as colorimetry, enzyme-linked immunosorbent assays (ELISA), and high-performance liquid chromatography (HPLC) have been commonly employed for this purpose [15]. However, these methods often require expensive equipment, skilled personnel, and time-consuming sample preparation procedures, which limit their applicability for routine monitoring [16,17]. As a result, researchers have been exploring more advanced technologies, including chemiluminescence, fluorescence, surface-enhanced Raman scattering (SERS), and electrochemical sensors, all of which offer the potential for faster, more cost-effective, and sensitive detection [18,19,20]. Among these technologies, electrochemical sensors, particularly ECL biosensors, have emerged as promising alternatives owing to their high sensitivity, simple operation, real-time analysis, and minimal sample preparation [21,22]. As a classic analytical technique originally established by pioneering researchers from the United States and Europe, ECL produces light via electrochemical reactions, enabling the highly sensitive detection of trace analytes. With outstanding advantages of low background, high accuracy, and reliable quantitative ability, ECL biosensors have been widely used in environmental monitoring and biomedical applications [23,24]. In these sensors, the chemiluminescent signal is generated by the electrochemical oxidation or reduction of a luminophore, often facilitated by a co-reactant that enhances the reaction [25,26]. One of the key challenges in designing efficient ECL biosensors is the choice of suitable co-reactant materials, as they play a critical role in controlling the intensity and stability of the emitted light. Metal nanocatalysts, especially those with a unique two-dimensional (2D) structure, have emerged as highly promising co-reactants in ECL systems [18,27]. These materials, including transition metal alloys and composites, exhibit superior catalytic properties, high surface areas, and low toxicity, making them ideal candidates for use in ECL biosensors [28,29]. The high surface area of metal nanocatalysts enables enhanced interaction with the electrochemical reactants, while their unique electronic properties facilitate the efficient transfer of electrons during the reaction process. Additionally, the low toxicity of these materials ensures that the biosensors can be used in biological and environmental samples without introducing harmful effects. Recent studies have demonstrated that two-dimensional nanomaterials and metal-based composites can significantly enhance ECL biosensor performance. Metallenes are atomically thin two-dimensional metallic nanosheets composed of closely packed single or few atomic layers of metals or alloys. They feature a purely metallic framework with a continuous metallic two-dimensional structure devoid of non-metal supports. These materials exhibit an ultrathin sheet-like morphology, high specific surface area, abundant surface active sites, excellent electrical conductivity, and tunable multimetallic synergy. Compared with graphene, MoS_2_, MXene, and MOF-derived two-dimensional materials, metallenes offer distinctive advantages, including fully exposed active sites, high metallic conductivity, readily tunable electronic structures, robust structural stability, and fast charge transport. For instance, programmable gold nanoclusters (AuNCs) gel spheres have been used as efficient ECL emitters to construct sensitive biosensors [30]. Additionally, ruthenium–copper metal–organic frameworks (RuCu MOFs) have been employed in a signal on-off-on ECL aptasensor strategy, enabling highly sensitive detection of this hepatotoxin [31]. Moreover, the integration of CRISPR-Cas12a with reactive oxygen species (ROS)-responsive luminophores has led to dual-mode biosensing platforms with enhanced signal amplification [32]. The use of spatially separated sensing and reporting architectures has also been shown to reduce interference between the target analyte and luminescent species, thereby improving detection accuracy [33,34,35].

In this work, a high-performance ECL biosensing platform based on PdMoCu trimetallic metallenes was developed for ultrasensitive MC-LR detection. The PdMoCu metallenes, synthesized via a controllable wet-chemical method, possess ultrathin 2D nanostructures with abundant active sites and excellent electronic properties. By integrating the metallenes with an aptamer-regulated DNA interface and Ru(bpy)_3_^2+^ ECL system, a signal-switchable sensor was constructed, achieving an ultralow femtogram-per-milliliter detection limit, a wide linear range and good reliability in real water samples. Benefiting from the synergistic electronic modulation of trimetallic metallenes, the platform outperforms conventional ECL systems with accelerated charge transfer and enhanced signal stability. Although mono-/bimetallic metallenes have been studied, few reports use PdMoCu trimetallic metallenes to enhance Ru(bpy)_3_^2+^/TPrA ECL with DNA electrostatic enrichment and aptamer signal switching. This work constructs a PdMoCu catalytic interface and programmable H1–aptamer duplex, enabling label-free, enzyme-free femtogram-level MC-LR detection with high selectivity, offering a universal strategy for ultrasensitive environmental toxin monitoring. This work not only provides a sensitive method for environmental toxin monitoring but also expands the application of metal-based 2D nanomaterials in ECL bioanalysis, offering a general framework for trace contaminant detection. Furthermore, the platform shows great potential in early cancer diagnosis and precision medicine, enabling the detection of trace disease-related molecules via aptamer recognition and ECL signal amplification, and promoting the expansion of biosensing technology to clinical precision medicine.

## 2. Materials and Methods

A list of all reagents and instruments used in this experiment is presented in the Supplementary Materials.

### 2.1. Synthesis of PdMoCu Trimetallic Nanocatalysts

The PdMoCu trimetallic metallenes were synthesized through a controlled wet-chemical method involving precursor coordination, ultrasonic dispersion, and low-temperature solvothermal reaction. Briefly, three clean and dry 50 mL beakers were prepared and labeled A, B, and C. Each beaker was charged with 50.0 mg palladium acetylacetonate (Pd(acac)_2_) (Sinopharm Chemical Reagent Co., Ltd., Shanghai, China) and 100.0 mg molybdenum hexacarbonyl (Mo(CO)_6_) (Sinopharm Chemical Reagent Co., Ltd., Shanghai, China). Copper acetylacetonate (Cu(acac)_2_) (Sinopharm Chemical Reagent Co., Ltd., Shanghai, China) was then added at different molar ratios relative to Pd (Cu/Pd = 0.1, 0.5, and 1, respectively) to regulate the trimetallic composition. Subsequently, 40 mL of anhydrous N,N-dimethylformamide (DMF) (Sinopharm Chemical Reagent Co., Ltd., Shanghai, China) was introduced into each system as the reaction solvent. The mixtures were sealed and ultrasonically treated at 25 ± 2 °C with a power of 300 W for 30 min to obtain homogeneous transparent light-green precursor solutions, with color variation corresponding to Cu content. The solutions were then transferred into 100 mL round-bottom flasks, followed by the addition of 10 mL glacial acetic acid (Macklin Biochemical Co., Ltd., Shanghai, China) as a coordinating and structure-directing agent. The reaction was conducted in an oil bath at 50 ± 0.5 °C for 12 h, during which black precipitates gradually formed, indicating the generation of PdMoCu metallenes. After cooling to room temperature, the products were collected by centrifugation at 7500 rpm for 10 min. The precipitates were thoroughly purified through repeated washing (five cycles) with a cyclohexane/ethanol mixed solvent (1:1, *v*/*v*), each cycle consisting of ultrasonic redispersion followed by centrifugation to remove unreacted precursors and organic residues. Finally, the purified materials were vacuum-dried at 50 °C for 6 h to obtain the PdMoCu trimetallic metallene powders for subsequent characterization and sensor fabrication (DNA was obtained from Sangon Biotech (Shanghai) Co., Ltd. (Shanghai, China). BSA was obtained from Thermo Fisher Scientific Inc. (Waltham, MA, USA). Antigens and antibodies were obtained from Aladdin Biochemical Technology (Shanghai) Co., Ltd. Shanghai, China).

### 2.2. DNA Preparation and Biosensor Construction

The DNA hairpin (H1) structure was formed by incubating the DNA at 95 °C for 10 min followed by rapid cooling. The ECL DNA biosensor was constructed through a stepwise surface modification strategy on a glassy carbon electrode (GCE). Prior to modification, the GCE was carefully polished with alumina slurry to obtain a smooth and clean surface, followed by sequential ultrasonic cleaning in ultrapure water and ethanol to remove residual particles and organic contaminants. The electrochemical performance of the pretreated electrode was evaluated in a 5.0 mmol L^−1^ [Fe(CN)_6_]^3−^/^4−^ solution by cyclic voltammetry until the peak-to-peak potential separation (ΔEp) was less than 0.11 V, ensuring a well-activated and mirror-like conductive surface suitable for subsequent functionalization. After drying under ambient conditions, 6 μL of PdMoCu trimetallic metallene dispersion (1.5 mg mL^−1^) was drop-cast onto the electrode surface and allowed to dry naturally, forming a uniform catalytic layer. Subsequently, 6 μL of gold nanoparticles (Au NPs) was deposited to provide abundant Au–S binding sites for thiolated DNA immobilization. The modified electrode was then incubated with pretreated hairpin DNA (H1) solution for 30 min to enable stable anchoring via Au–S bonds. After rinsing with 0.1 M phosphate-buffered saline (PBS, pH 7.4) to remove unbound strands, 6 μL of 1 wt% bovine serum albumin (BSA) was applied to block nonspecific adsorption sites and minimize background interference. Thereafter, 10 μL of aptamer (Apt) solution was introduced to hybridize with H1, forming a double-stranded DNA structure on the electrode interface. For ECL signal generation, tris(2,2′-bipyridyl)ruthenium(II) hexahydrate was dissolved in PBS to prepare a 1 mg mL^−1^ Ru(bpy)_3_^2+^ solution (40 mg in 40 mL), and an appropriate volume was drop-coated onto the electrode surface to allow electrostatic adsorption within the negatively charged DNA duplex. Finally, 10 μL of MC-LR solutions with varying concentrations were incubated on the modified electrode to enable specific recognition by the aptamer, thereby completing the fabrication of the ECL DNA biosensor for subsequent analytical measurements.

Aptamer: GGGCGTGGCTAGAAAAACACGCCCTATTC.

### 2.3. ECL Measurements

The performance evaluation of the ECL biosensor was carried out using a combined electrochemical workstation and an ultra-weak luminescence/bioluminescence detection system to enable simultaneous electrochemical excitation and photonic signal acquisition. A conventional three-electrode configuration was employed, in which the modified GCE served as the working electrode, a platinum wire acted as the counter electrode, and an Ag/AgCl electrode was used as the reference electrode. The electrolyte consisted of phosphate-buffered saline (PBS) at a concentration of 1/15 mmol L^−1^, providing a stable ionic environment for the ECL reaction. Cyclic voltammetry (CV) was performed within a potential window of 0–0.8 V to trigger the redox reactions of the Ru(bpy)_3_^2+^/co-reactant system and generate the corresponding chemiluminescence signal. To optimize analytical performance, the effects of solution pH and Ru(bpy)_3_^2+^ incubation time on ECL intensity were systematically investigated, and the optimal experimental parameters were determined based on maximum signal output and signal stability. Under the optimized conditions, a series of analyte solutions with varying concentrations was incubated on the sensor surface, and their corresponding ECL responses were recorded. Calibration curves were established by plotting ECL intensity against the logarithm of analyte concentration, and the limit of detection (LOD) was calculated according to the signal-to-noise ratio (S/N = 3) criterion, thereby quantitatively evaluating the sensitivity and analytical capability of the constructed biosensor.

## 3. Results

### 3.1. The Principle for ECL Detection of MC-LR

**Scheme 1:**
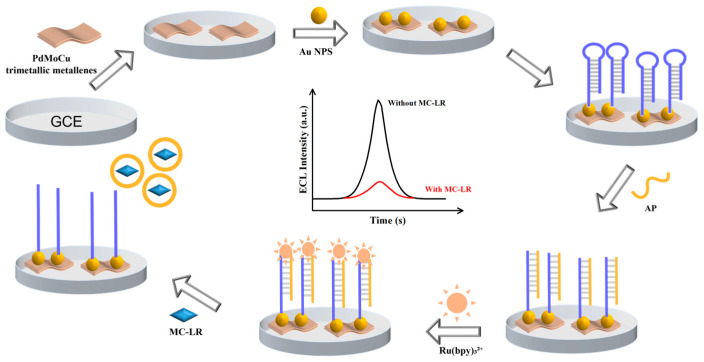
The principle for ECL detection of MC-LR using PdMoCu trimetallic metallene.

The sensing mechanism of the proposed platform is schematically illustrated in Scheme 1 and is based on a metallene-enhanced interfacial ECL system coupled with target-triggered DNA structural switching. The overall design integrates catalytic interface engineering, electrostatic luminophore enrichment, and aptamer-mediated molecular recognition into a unified signal-transduction framework. The PdMoCu trimetallic metallene layer serves as the fundamental electroactive interface. Owing to its ultrathin two-dimensional architecture and synergistic electronic coupling among Pd, Mo, and Cu atoms, the metallene significantly accelerates interfacial charge-transfer kinetics and lowers the energy barrier for the Ru(bpy)_3_^2+^ oxidation process. The enlarged electrochemically active surface area further promotes luminophore accessibility, thereby amplifying the intrinsic ECL response. Subsequent immobilization of thiolated hairpin DNA (H1) onto Au nanoparticle-decorated surfaces establishes a programmable molecular scaffold. Hybridization between H1 and the MC-LR aptamer forms a rigid double-stranded DNA architecture enriched with negatively charged phosphate backbones. This polyanionic interface acts as an electrostatic reservoir, effectively concentrating positively charged Ru(bpy)_3_^2+^ species near the electrode surface. As a result, a high local luminophore density is achieved, leading to an intensified ECL emission under potential scanning. Upon introduction of MC-LR, specific aptamer–target binding induces a conformational transition of the aptamer strand, destabilizing the duplex structure. The disruption of the double-stranded DNA framework weakens the electrostatic confinement effect, resulting in partial release of interfacially adsorbed Ru(bpy)_3_^2+^. Consequently, the effective surface concentration of the luminophore decreases. During cyclic voltammetric excitation, Ru(bpy)_3_^2+^ is oxidized to Ru(bpy)_3_^3+^, which reacts with the co-reactant radical to generate the excited state Ru(bpy)_3_^2+^*. The radiative decay of this excited species produces the measurable ECL emission. Because the ECL intensity is directly dependent on the interfacial luminophore population and charge-transfer efficiency, the target-induced luminophore depletion results in a proportional attenuation of the emission signal. Therefore, the sensor operates via a signal-off mechanism in which ECL intensity inversely correlates with MC-LR concentration. The metallene-enhanced electron-transfer dynamics synergistically amplify signal variation, enabling ultrasensitive quantification across a broad dynamic range. In essence, the analytical performance arises from the cooperative interplay of (i) catalytic electron-transfer acceleration by PdMoCu metallenes, (ii) DNA duplex-mediated electrostatic luminophore enrichment, and (iii) aptamer-triggered structural switching for controllable signal modulation. This integrated interfacial engineering strategy establishes a robust and highly sensitive ECL platform for trace-level MC-LR detection.

### 3.2. Materials Characterization

**Figure 1:**
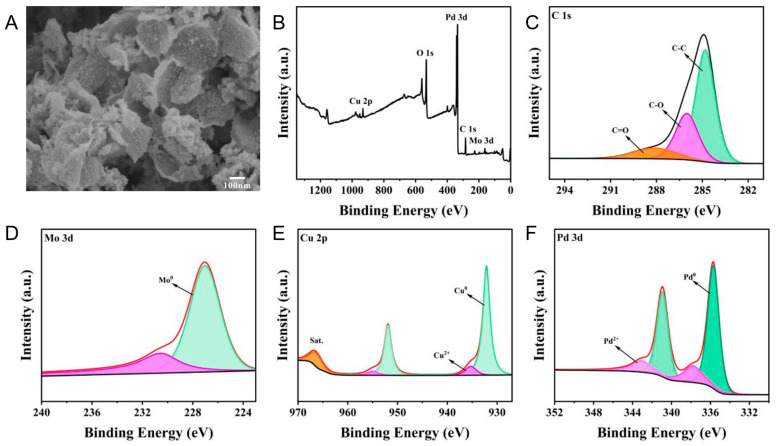
(**A**) SEM image of PdMoCu trimetallic metallenes. (**B**) XPS survey, (**C**) C 1s, (**D**) Mo 3d, Cu 2p (**E**), and Pd 3d (**F**) spectrum of PdMoCu trimetallic metallenes.

SEM characterization revealed that the as-synthesized PdMoCu trimetallic metallenes presented a typical wrinkled 2D ultrathin nanosheet morphology with good lateral continuity (Figure 1A). The TEM images clearly show that the PdMoCu metallene exhibits a typical two-dimensional wrinkled nanosheet structure with good lateral continuity (Figure S1). This sheet-like structure indicates effective inhibition of vertical crystal growth during wet-chemical synthesis, which arises from synergistic lattice strain redistribution caused by Cu doping and ligand-mediated anisotropic growth. The ultrathin structure provides abundant surface active sites and short electron-transport pathways, favoring interfacial charge transfer and ECL signal amplification.

To further elucidate the surface chemical states and elemental composition, X-ray photoelectron spectroscopy (XPS) analysis was performed. The survey spectrum confirms the coexistence of Pd, Mo, Cu, C, and O elements, indicating successful incorporation of all three metallic components within the hybrid structure. The high-resolution C 1s spectrum displays characteristic peaks at 284.8, 286.1, and 288.1 eV, corresponding to C–C, C–O, and C=O species, respectively, which originate from residual organic ligands and surface-adsorbed species during synthesis(Figure 1B,C).

The Mo 3d spectrum reveals two dominant peaks at 227.1 and 230.6 eV, assignable to metallic Mo^0^ (3d_5_/_2_ and 3d_3_/_2_), confirming the presence of reduced molybdenum species. In the Cu 2p region, characteristic peaks at 932.2 and 952.0 eV are attributed to Cu^0^ (2p_3_/_2_ and 2p_1_/_2_), while additional signals at 935.4 and 954.9 eV, accompanied by a satellite feature at 966.8 eV, indicate the coexistence of Cu^2+^ species. This mixed-valence state suggests partial surface oxidation and potential electronic interaction between Cu and neighboring metal atoms. The Pd 3d spectrum predominantly exhibits peaks at 335.7 and 340.9 eV, corresponding to Pd^0^ species, whereas weaker signals at 337.8 and 343.1 eV indicate minor Pd^2+^ formation due to surface oxidation upon air exposure (Figure 1D,E).

The coexistence of multiple metallic states and partial surface oxidation indicates electronic redistribution among Pd, Mo, and Cu atoms, giving rise to interfacial charge polarization and lattice strain—key factors for catalytic enhancement. XPS results thus verify the successful synthesis of PdMoCu trimetallic metallenes and disclose their electronically interactive surface, which markedly promotes interfacial electron transfer and boosts ECL emission. Under identical conditions, the ECL signal was weak with only AuNPs owing to slow electron transfer, but was significantly enhanced after introducing PdMoCu metallene (Figure S2). The ECL signal intensity of the PdMoCu trimetallic system is significantly superior to that of mono- and bimetallic systems, demonstrating the critical role of the trimetallic synergistic effect in enhancing catalytic efficiency (Figure S3). Although AuNPs are widely utilized due to their excellent electrical conductivity and abundant surface sites favorable for DNA immobilization, their catalytic efficiency in the Ru(bpy)_3_^2+^/TPrA system remains inferior to that of our PdMoCu trimetallene. Specifically, AuNPs typically rely on physical adsorption or simple electron transfer mediation to enhance the ECL signal, which often results in moderate catalytic activity and limited signal amplification capability. In contrast, the PdMoCu metallene leverages synergistic electronic modulation among the three metals to significantly accelerate interfacial charge transfer and reduce the reaction energy barrier. Consequently, the ECL intensity induced by PdMoCu is substantially higher than that generated by AuNPs, clearly demonstrating the superior catalytic performance and unique advantages of the trimetallic structure in enhancing ECL emission.

### 3.3. Electrochemical Impedance Analysis of Interfacial Assembly

**Figure 2:**
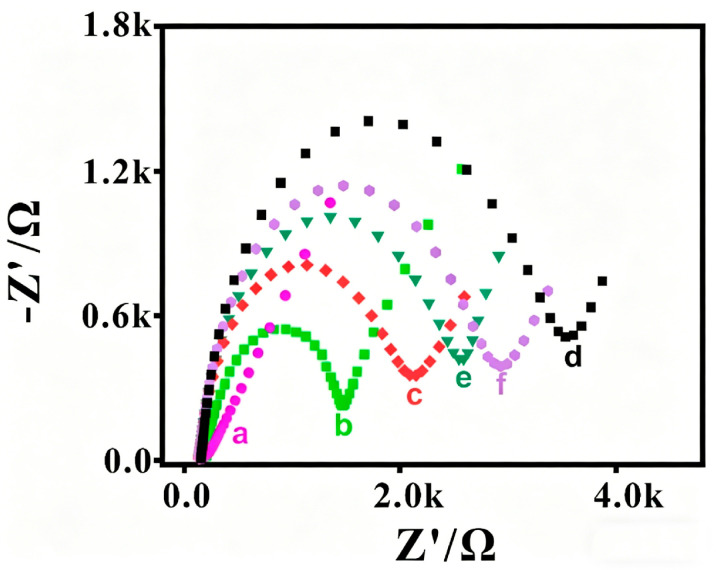
Electrochemical impedance spectra: (a) bare electrode; (b) PdMoCu trimetallic metallenes; (c) PdMoCu trimetallic metallenes/H1; (d) PdMoCu trimetallic metallenes/H1/Apt; (e) PdMoCu trimetallic metallenes/H1/Apt/Ru(bpy)_3_^2+^; (f) PdMoCu trimetallic met-allenes/H1/Apt/Ru(bpy)_3_^2+^/MC-LR.

Electrochemical impedance spectroscopy (EIS) was used to monitor the stepwise assembly of the biosensor and characterize interfacial charge-transfer behavior (Figure 2). Tests were performed in a three-electrode system with 2.5 mmol L^−1^ [Fe(CN)_6_]^3−^/^4−^ containing 0.1 mol L^−1^ KCl as the redox probe, where the semicircle diameter represents the charge-transfer resistance (R_ct_). The bare glassy carbon electrode showed a small R_et_, indicating fast electron transfer. Deposition of PdMoCu metallenes moderately increased R_et_ by forming a nanostructured layer while preserving good conductivity. Subsequent immobilization of H1 and aptamer hybridization sharply raised R_ct_, as negatively charged DNA impeded the redox probe diffusion, confirming successful DNA duplex assembly. Adsorption of positively charged Ru(bpy)_3_^2+^ reduced R_ct_ by neutralizing surface negative charges and verifying effective electrostatic enrichment. Finally, incubation with MC-LR increased R_ct_ again, because aptamer–target binding dissociated the DNA duplex and released Ru(bpy)_3_^2+^. The sequential R_ct_ changes fully validate the stepwise fabrication and target-responsive interfacial mechanism (Table S1). The apparent electron transfer rate constant (ks) was calculated to evaluate the interfacial electron transfer kinetics of the PdMoCu metallene-modified GCE and bare GCE. Cyclic voltammetry (CV) measurements were performed at different scan rates (0.02, 0.05, 0.1, 0.2 and 0.5 V/s) in the Ru(bpy)_3_^2+^/TPrA system. Based on the Laviron equation (for quasi-reversible reaction), the oxidation peak potential (Epa) was plotted against the logarithm of scan rate (logv), and a good linear relationship was obtained with a correlation coefficient R^2^ > 0.98. The charge transfer coefficient (α) was derived from the slope of the linear fit, and then the ks value was calculated by the intercept of the linear equation. The ks of PdMoCu metallene-modified GCE was ~6.26 × 10^−1^ cm·s^−1^, which was much higher than that of bare GCE (~5.0 × 10^−3^ cm·s^−1^), indicating that the PdMoCu trimetallic metallenes significantly accelerated the interfacial electron transfer kinetics via synergistic electronic modulation. Quantitative analysis of DNA loading and hybridization efficiency on the electrode surface was conducted using electrochemical methods. The surface density of hairpin DNA H1 was determined to be 5.26 × 10^−11^ mol·cm^−2^, and the hybridization efficiency between H1 and the aptamer reached 87.3%. These results indicate that DNA immobilization on the electrode surface is efficient and hybridization efficiency is high, demonstrating that the sensing interface is assembled uniformly, stably, and controllably. Overall, the sequential variation in charge-transfer resistance clearly demonstrates the successful stepwise fabrication of the sensing platform and corroborates the target-responsive interfacial modulation mechanism. The impedance evolution is fully consistent with the proposed signal-transduction pathway, confirming the reliability of the constructed ECL biosensor.

### 3.4. Optimization of Experimental Parameters

To achieve maximal analytical performance, key interfacial and reaction parameters influencing the ECL response were systematically optimized, including the pH of the supporting electrolyte and the adsorption time of Ru(bpy)_3_^2+^ on the DNA-modified interface. Prior to the actual detection of complex high-salt samples, appropriate dilution or buffer adjustment is generally required to maintain the efficiency of electrostatic enrichment.

The pH of the phosphate-buffered saline plays a critical role in modulating both the electrochemical behavior of the Ru(bpy)_3_^2+^/co-reactant system and the structural stability of the DNA duplex layer. As shown in Figure S4, the ECL intensity gradually increases as the pH is adjusted from 6.0 to 7.4, reaching a maximum at physiological pH. This enhancement can be attributed to improved co-reactant oxidation efficiency and favorable proton-coupled electron-transfer kinetics under near-neutral conditions. Moreover, at pH 7.4, the DNA duplex maintains optimal structural stability and surface charge density, thereby maximizing electrostatic enrichment of Ru(bpy)_3_^2+^. When the pH is further increased to 7.8, a noticeable decline in ECL intensity is observed. This decrease may result from partial alteration of the DNA interfacial conformation and changes in the protonation state of the co-reactant, which collectively affect luminophore oxidation efficiency and excited-state generation. Therefore, pH 7.4 was selected as the optimal operating condition for subsequent experiments, ensuring balanced interfacial stability and electron-transfer kinetics.

The adsorption time of Ru(bpy)_3_^2+^ directly determines the degree of electrostatic accumulation of the luminophore within the negatively charged DNA duplex framework, thereby influencing the effective surface concentration and ECL output. As illustrated in Figure S5, the ECL intensity increases progressively with extended adsorption time up to 60 min, indicating gradual establishment of adsorption equilibrium and increasing interfacial luminophore density. Beyond 60 min, the ECL signal reaches a plateau, suggesting that surface binding sites become saturated and the system approaches dynamic equilibrium. Prolonged incubation does not yield further signal enhancement, indicating that maximal electrostatic enrichment has been achieved. Accordingly, 60 min was selected as the optimal adsorption time, balancing signal intensity and experimental efficiency.

Overall, these optimization results demonstrate that the ECL performance of the biosensor is highly dependent on controlled interfacial kinetics and physicochemical conditions. Precise regulation of electrolyte pH and luminophore adsorption equilibrium ensures maximal signal amplification and reproducibility in subsequent analytical measurements.

### 3.5. Sensitivity Evaluation of the ECL Biosensor

**Figure 3:**
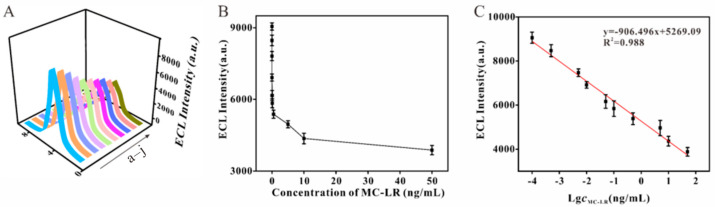
Analytical performance of the ECL biosensor for MC-LR detection. (**A**) ECL emission responses obtained at increasing MC-LR concentrations (a–j correspond to 0.1 pg mL^−1^, 0.5 pg mL^−1^, 5 pg mL^−1^, 10 pg mL^−1^, 50 pg mL^−1^, 100 pg mL^−1^, 500 pg mL^−1^, 5 ng mL^−1^, 10 ng mL^−1^, and 50 ng mL^−1^, respectively). (**B**) Dependence of ECL intensity on MC-LR concentration. (**C**) Linear calibration curve between ECL intensity and the logarithm of MC-LR concentration (n = 3).

The analytical sensitivity of the constructed ECL biosensor was systematically evaluated under the optimized experimental conditions. As illustrated in Figure 3A, the ECL emission profiles were recorded at increasing concentrations of MC-LR. With the gradual increase in MC-LR concentration, the ECL intensity progressively decreased, indicating a concentration-dependent signal attenuation behavior. This trend is consistent with the proposed signal-off sensing mechanism, in which target binding induces partial release of surface-confined Ru(bpy)_3_^2+^ and reduces the effective luminophore density at the electrode interface. Figure 3B further presents the variation in ECL intensity as a function of MC-LR concentration. A clear monotonic decrease in emission intensity was observed as the concentration increased from 0.1 pg mL^−1^ to 50 ng mL^−1^, demonstrating the sensor’s capability to transduce molecular recognition events into quantitative optical signals over a wide concentration range. As shown in Figure 3C, a well-defined linear relationship was established between the ECL intensity (IECL) and the logarithm of MC-LR concentration over a broad dynamic range from 0.1 pg mL^−1^ to 50 ng mL^−1^. The corresponding linear regression equation was expressed as IECL = 5269.09 − 906.496 log c, with a correlation coefficient (R^2^) of 0.988, indicating good linearity and reliable quantitative performance. The slope of the calibration curve reflects the intrinsic analytical sensitivity of the platform. Such a high signal–response gradient originates from the synergistic amplification mechanisms embedded in the sensing architecture. Specifically, the ultrathin PdMoCu metallene layer accelerates interfacial electron-transfer kinetics and enhances the efficiency of Ru(bpy)_3_^2+^ oxidation, while the DNA duplex-mediated electrostatic enrichment significantly increases the local luminophore concentration. The combination of catalytic enhancement and target-triggered luminophore depletion generates a pronounced signal variation even at ultralow MC-LR concentrations. Based on the signal-to-noise ratio criterion (S/N = 3), the limit of detection (LOD) was calculated to be 37 fg mL^−1^. The formula is LOD = 3σ/k, where σ represents the standard deviation of 11 repeated measurements of blank samples, and k is the slope of the calibration curve. The noise level (σ) was determined as the standard deviation of ECL intensities from 11 repeated measurements of the blank solution without MC-LR. This femtogram-level detection capability demonstrates the remarkable sensitivity of the proposed strategy. The exceptionally low LOD can be attributed to three cooperative factors: (i) efficient metallene-mediated charge-transfer acceleration, (ii) high-density surface confinement of Ru(bpy)_3_^2+^ via electrostatic interaction, and (iii) structurally amplified signal attenuation induced by aptamer-target binding. We also measured the ECL signal near the limit of detection (LOD). The results demonstrate that the relative standard deviation (RSD) near the LOD remains at a low level, verifying the reliability of the sensing platform at ultra-trace concentrations (Figure S6).

Based on the Langmuir adsorption isotherm model $\frac{\Delta {ECL}_{Intensity}}{{{ECL}_{Intensity}}_{0}}=\frac{\Delta {ECL}_{max}\cdot C}{K_{d}+C}$, we performed nonlinear fitting on the calibration data of MC-LR concentration versus ECL response, and obtained the key parameters of interfacial binding and sensing performance. The fitting results showed that the maximum relative signal change ΔECLmax/ECLIntensity0 was 0.92 ± 0.03, which was highly consistent with the saturated response observed in experiments, representing the maximum signal attenuation achievable by the sensor. Meanwhile, the apparent dissociation constant of the aptamer-MC-LR interaction was fitted as Kd = (1.2 ± 0.2) × 10^−11^ mol/L (corresponding to a mass concentration of approximately 0.01 ng/mL). This extremely low K d value fully demonstrates the high-affinity binding property between the aptamer and the target, verifying the specific recognition capability of the sensing interface. In addition, the goodness of fit of the model was R^2^ = 0.991, indicating that the Langmuir model can accurately describe the equilibrium relationship between ECL response and MC-LR concentration over a wide concentration range of 0.1 pg/mL to 50 ng/mL, further confirming that the sensing process is governed by molecular recognition equilibrium.

Importantly, the achieved sensitivity is competitive with, and in many cases superior to, previously reported ECL or electrochemical sensing platforms for MC-LR detection (Table S2). Although CRISPR offers extremely high specificity, this work achieves enzyme-free ultrasensitive detection through the intrinsic catalytic activity of PdMoCu metallenes, which significantly simplifies the operation procedure and shortens the assay time. The wide linear range spanning over five orders of magnitude further highlights the robustness and adaptability of the system for trace-level environmental monitoring. Overall, the combination of broad dynamic range, high signal amplification efficiency, and femtogram-scale detection limit confirms that the proposed ECL biosensor possesses outstanding analytical sensitivity for MC-LR determination.

### 3.6. Specificity and Anti-Interference Performance

**Figure 4:**
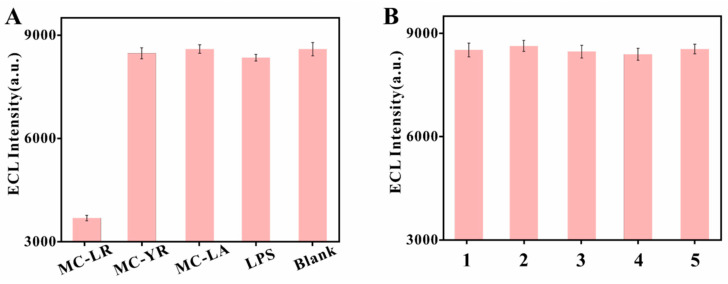
Evaluation of selectivity and reproducibility of the ECL biosensor. (**A**) Selectivity assessment of the sensor toward MC-LR (50 ng mL^−1^) in the presence of potential interfering substances, including MC-YR, MC-LA, LPS, and blank control. (**B**) Reproducibility evaluation based on five independent measurements using the fabricated biosensor under identical conditions.

The selectivity of the proposed ECL biosensor toward MC-LR was systematically evaluated by examining its response to structurally related analogues and potential interfering species. Microcystin-YR (MC-YR), microcystin-LA (MC-LA), lipopolysaccharide (LPS), and a blank control were introduced into the analytical system under identical experimental conditions. The ECL responses were recorded and compared to those of MC-LR at the same concentration level. As shown in Figure 4A, a pronounced decrease in ECL intensity was observed exclusively in the presence of MC-LR, whereas the signals obtained for MC-YR, MC-LA, LPS, and the blank group exhibited negligible variation relative to the control. Despite the structural similarity between MC-LR and other microcystin analogues, the sensor demonstrated strong discrimination capability, indicating that the aptamer possesses high binding affinity and sequence-specific recognition toward MC-LR. This result confirms that minor differences in amino acid residues among microcystin variants do not trigger significant conformational switching of the aptamer unless MC-LR is present. In the selectivity experiments, lipopolysaccharide (LPS) was introduced as a representative biological macromolecule interferent (Figure S7). The results showed that LPS exerted negligible effects on the ECL signal, which confirms the effectiveness of the specific recognition by the aptamer and the BSA blocking layer in reducing nonspecific adsorption. The minimal signal fluctuation observed for non-target species suggests that nonspecific adsorption and cross-reactivity are effectively suppressed. This can be attributed to the dual-regulation mechanism embedded in the sensing interface: (i) the high specificity of aptamer–target molecular recognition and (ii) BSA-mediated blocking of nonspecific binding sites on the electrode surface. Together, these factors contribute to reduced background interference and enhanced signal fidelity. Furthermore, the ability to differentiate MC-LR from structurally homologous toxins highlights the robustness of the DNA-based recognition strategy and confirms the reliability of the signal-transduction mechanism. The excellent anti-interference performance suggests that the proposed biosensor is well-suited for practical applications in complex environmental matrices. Overall, the distinct signal response toward MC-LR in comparison with other potential interferents demonstrates the high selectivity and molecular specificity of the developed ECL biosensing platform.

### 3.7. Reproducibility and Stability Evaluation

To verify its analytical reliability, the reproducibility and operational stability of the constructed ECL biosensor were systematically evaluated. To further examine the fabrication reproducibility (inter-electrode precision), five independently prepared electrodes were tested under identical conditions. As shown in Figure 4B, the resulting ECL responses were highly consistent, with minimal variation among different sensor batches. The low RSD value confirms that the layer-by-layer assembly strategy provides a reliable and controllable surface modification. The ECL signal of the sensor remains nearly unchanged with negligible attenuation over 7 days of storage at 4 °C, demonstrating its excellent long-term stability (Figure S8).

Operational stability was assessed through continuous cyclic voltammetric scanning of the modified electrode. As presented in Figure S9, the recorded ECL intensity exhibited negligible fluctuation during repeated scans, indicating stable signal output upon successive electrochemical excitation. The calculated relative standard deviation (RSD) was 2.37%, demonstrating excellent measurement precision and minimal signal drift. This stable performance suggests that the PdMoCu metallene layer maintains robust interfacial conductivity and structural integrity, while the DNA duplex framework preserves its surface organization without significant desorption or degradation during electrochemical operation. The excellent reproducibility and stability can be attributed to several factors: (i) strong Au–S bonding ensures stable DNA immobilization; (ii) BSA blocking minimizes non-specific adsorption and surface fouling; and (iii) the metallene-enhanced interface provides durable electron-transfer pathways with reduced structural collapse. Overall, the low RSD values and consistent signal retention confirm that the developed ECL biosensor possesses high reliability, structural robustness, and operational durability, making it well-suited for practical environmental monitoring applications.

Due to the irreversible nature of the specific aptamer-target binding and DNA duplex dissociation, this sensor is designed for disposable use to ensure the highest detection accuracy. Meanwhile, the facile polishing and re-modification of GCEs offer feasibility for practical, low-cost and reproducible fabrication.

### 3.8. Real Sample Analysis and Practical Applicability

To evaluate the practical applicability of the developed ECL biosensor, real sample analysis was performed using tap water as a representative environmental matrix. Standard addition experiments were conducted by spiking known concentrations of MC-LR into tap water samples, followed by detection under the optimized experimental conditions. This approach was employed to assess the analytical accuracy, precision, and matrix tolerance of the sensing platform. As summarized in Table 1, the recoveries for MC-LR at different spiking levels ranged from 99.3% to 102.0%, indicating excellent analytical accuracy. The corresponding relative standard deviations (RSDs) varied from 1.1% to 3.6%, demonstrating satisfactory precision and reproducibility in complex sample matrices. The recovery values close to 100% confirm that the sensor response is not significantly affected by coexisting ions or background constituents present in tap water. The minimal matrix interference observed in real sample analysis can be attributed to the high specificity of the aptamer–target interaction and the effective suppression of nonspecific adsorption by surface blocking strategies. In addition, the metallene-enhanced ECL interface maintains stable electron-transfer performance even in the presence of potential interfering substances, ensuring consistent signal output. These results collectively demonstrate that the proposed biosensor possesses strong anti-matrix interference capability and reliable quantitative performance in practical environmental samples. The excellent recovery and low variability suggest that the developed platform holds significant potential for real-world monitoring of trace MC-LR contamination in drinking water and other aquatic environments.

### 3.9. Mechanistic Investigation of the ECL Process

To elucidate the signal-generation pathway of the proposed biosensing platform, the ECL mechanism was analyzed based on electrochemical behavior, optimization results, and the established signal-transduction model. The ECL emission originates from the classical Ru(bpy)_3_^2+^/tripropylamine (TPrA) co-reactant system, while its efficiency is significantly modulated by the PdMoCu metallene-enhanced interface and DNA-mediated luminophore enrichment.

Under positive potential scanning, TPrA is first oxidized at the electrode surface to generate the radical cation TPrA•^+^:

(1) $$TPrA-e^{-}\to TPr{A•}^{+}$$

The radical cation subsequently undergoes deprotonation to form the strongly reducing radical species TPrA•:

(2) $$\mathrm{TPrA}{•}^{+}-e^{-}\to \mathrm{TPrA}•+H^{+}$$

Simultaneously, Ru(bpy)_3_^2+^ is oxidized to Ru(bpy)_3_^3+^:

(3) $${\mathrm{Ru}(\mathrm{bpy})}_{3}^{2+}-e^{-}\to {\mathrm{Ru}(\mathrm{bpy})}_{3}^{3+}$$

The highly reactive TPrA• radical then reacts with Ru(bpy)_3_^3+^ via a reductive pathway to generate the excited-state species Ru(bpy)_3_^2+^*:

(4) $${\mathrm{Ru}(\mathrm{bpy})}_{3}^{3+}+\mathrm{TPrA}•\to {\mathrm{Ru}(\mathrm{bpy})}_{3}^{2+*}$$

Finally, the excited luminophore returns to its ground state with photon emission:

(5) $${\mathrm{Ru}(\mathrm{bpy})}_{3}^{2+*}\to {\mathrm{Ru}(\mathrm{bpy})}_{3}^{2+}+h\nu$$

While the above reactions represent the conventional ECL pathway, the present system exhibits enhanced emission efficiency due to interfacial regulation by PdMoCu metallenes. The ultrathin metallene layer accelerates electron-transfer kinetics, lowers the overpotential for both TPrA and Ru(bpy)_3_^2+^ oxidation, and increases the effective collision probability between Ru(bpy)_3_^3+^ and TPrA•. The synergistic electronic coupling among Pd, Mo, and Cu atoms likely modifies the local electronic density of states, facilitating faster charge exchange at the electrode–electrolyte interface.

Moreover, the negatively charged DNA duplex acts as an electrostatic confinement matrix, enriching Ru(bpy)_3_^2+^ near the electrode surface and thereby increasing the local concentration of luminophore available for excitation. This confinement effect enhances the quantum efficiency of the ECL process by promoting spatial proximity between reactive intermediates. Upon binding of MC-LR, aptamer-induced duplex dissociation leads to partial release of Ru(bpy)_3_^2+^ from the interface. The decrease in interfacial luminophore density reduces the generation rate of Ru(bpy)_3_^2+^*, resulting in attenuated ECL emission. Therefore, the observed signal variation originates from the combined modulation of interfacial electron-transfer efficiency and luminophore availability.

Overall, the enhanced ECL response arises from a dual amplification mechanism: (i) catalytic acceleration of redox reactions by PdMoCu metallenes and (ii) electrostatic enrichment of Ru(bpy)_3_^2+^ via DNA duplex formation. The target-induced structural switching further converts molecular recognition events into measurable optical signals. This cooperative interfacial engineering mechanism underlies the ultrahigh sensitivity of the proposed MC-LR detection platform.

### 3.10. Future Perspectives

Despite the favorable analytical performance achieved in this work, practical applications in complex biological matrices still face challenges related to non-specific adsorption and high ionic strength. Notably, high ionic strength under physiological conditions may compromise the electrostatic enrichment of Ru(bpy)_3_^2+^ that underpins the sensing mechanism. To address these issues, future efforts will focus on integrating antifouling interface engineering strategies, such as the introduction of zwitterionic polymer coatings or rigid hydrophilic spacer layers, to suppress non-specific adsorption and shield the electrostatic interaction from interference by high salt concentrations. Further optimization of the DNA–aptamer duplex architecture and surface anchoring modes will also be explored to enhance structural stability and signal fidelity in serum and other biofluids, thereby extending the platform from environmental toxin monitoring toward clinical biosensing applications.

## 4. Conclusions

In summary, we have successfully developed a highly sensitive and reliable electrogenerated chemiluminescence (ECL) biosensing platform for the determination of microcystin-LR (MC-LR) by integrating PdMoCu trimetallic metallenes with a DNA-based molecular recognition interface. The ultrathin two-dimensional PdMoCu metallenes provided a catalytically active and electronically conductive surface, significantly enhancing interfacial charge-transfer kinetics and amplifying the Ru(bpy)_3_^2+^/TPrA ECL emission efficiency. Meanwhile, the programmable H1–aptamer duplex architecture enabled effective electrostatic enrichment of luminophore molecules and target-responsive structural switching, converting molecular recognition events into quantifiable optical signals. Under optimized conditions, the proposed sensor achieved a wide linear detection range from 0.1 pg mL^−1^ to 50 ng mL^−1^ with a correlation coefficient (R^2^) of 0.988 and an ultralow detection limit of 37 fg mL^−1^. The platform also demonstrated excellent selectivity toward MC-LR against structurally related analogues, as well as high reproducibility and operational stability with low relative standard deviations. In real tap water samples, satisfactory recoveries (99.3–102.0%) further confirmed its accuracy and strong anti-matrix interference capability. Mechanistically, the superior analytical performance originates from a synergistic interfacial engineering strategy involving metallene-accelerated redox kinetics, DNA-mediated luminophore confinement, and aptamer-triggered signal modulation. This cooperative amplification framework not only enhances ECL efficiency but also establishes a robust pathway for translating specific molecular recognition into amplified electrochemical optical outputs. Overall, this work expands the functional application of trimetallic metallenes in ECL biosensing and provides a versatile design strategy for constructing ultrasensitive detection platforms for trace environmental contaminants. The proposed methodology holds significant promise for real-time environmental monitoring and can be readily extended to the detection of other hazardous analytes through aptamer substitution and interface customization.

## Figures and Tables

**Table 1:** Application of the Sensor in Real Sample Analysis and Recovery.

Sample (ng/mL)	Add (ng/mL)	Detect (ng/mL)	RSD (%, *n* = 5)	Recovery (%)
Tap water	1.00	1.018	3.6	101.8
	10.00	10.04	1.5	100.4
	50.00	49.65	1.1	99.3

## Data Availability

The original contributions presented in the study are included in the article; further inquiries can be directed to the corresponding authors.

## Supplementary Materials

The following supporting information can be downloaded at: https://www.mdpi.com/article/10.3390/bios16050264/s1, Figure S1: TEM image of PdMoCu metallene. Figure S2: ECL Responses of Sensing Interfaces with and without PdMoCu Metallenes. Figure S3: Comparison of ECL performance among monometallic (Pd), bimetallic (PdMo), and trimetallic (PdMoCu) systems. Figure S4: Optimization of experimental PBS pH affecting the ECL response. Figure S5: Effect of Ru(bpy)_3_^2+^ adsorption time on the ECL response of the DNA-modified electrode. Figure S6: ECL signal near the LOD. Figure S7: Selectivity evaluation of the ECL sensing platform. Figure S8: Stability test of the biosensor over 7 days at 4 °C. Figure S9: The stability test of the proposed ECL sensor. Table S1: Quantitative characterization. Table S2: Comparison of the analytical performance of different sensors for MC-LR detection. References [30,31,36,37,38,39] are cited in Supplementary Materials.
