# Supplementary material for: Synergistic PdMoCu Trimetallic Metallene-Enhanced Electrochemiluminescence Biosensor for Ultrasensitive Detection of Microcystin-LR
Source: Biosensors (Basel). 2026 May 2;16(5):264. doi: 10.3390/bios16050264 (PMC13204624; doi:10.3390/bios16050264)
Supplement: Supplementary file 1 [file biosensors-16-00264-s001.zip › biosensors-4212486-supplementary.pdf]

Supporting Data

Materials and Instruments

The reagents used in this experiment include N,N-dimethylformamide (C_3H_7NO), palladium acetylacetonate ($Pd(acac)_2$), molybdenum hexacarbonyl ($Mo(CO)_6$), copper acetylacetonate, nickel acetylacetonate, anhydrous ethanol, glacial acetic acid, thioglycolic acid ($C_2H_4O_2S$), sodium chloride (NaCl), neuron-specific enolase antigen, neuron-specific enolase antibody, and bovine serum albumin (BSA). All these reagents are of analytical grade and were provided by manufacturers such as Sinopharm Chemical Reagent Co., Ltd., Macklin, Kermel, and Shanghai Genview Scientific Co., Ltd.

The main instruments employed in the experiment are an electrochemical workstation (RST5202F, Suzhou Shiruisi), an electric thermostatic blast vacuum drying oven (DZF-6020, Shanghai Boxun Industrial Co., Ltd.), a flow injection chemiluminescence detector (MPI-F, Xi'an Ruimai), an electronic analytical balance (AL204, Sartorius Scientific Instruments (Beijing) Co., Ltd.), an ultrasonic cleaner (KQ-100DE, Kunshan Ultrasonic Instrument Co., Ltd.), an X-ray diffractometer (Bruker D8, Bruker, Germany), a scanning electron microscope (FEI QUANTA FEG250, Oxford Instruments, UK), a high-speed microcentrifuge (D3024, Thermo Fisher Scientific Inc.), a pH meter (PHS-3B, Shanghai Precision & Scientific Instrument Co., Ltd.), an ultrapure water system (UPT-II-5T, Reax Electronic Technology Co., Ltd.), and a rapid mixer (SK-1, Zhongda Instrument Factory).

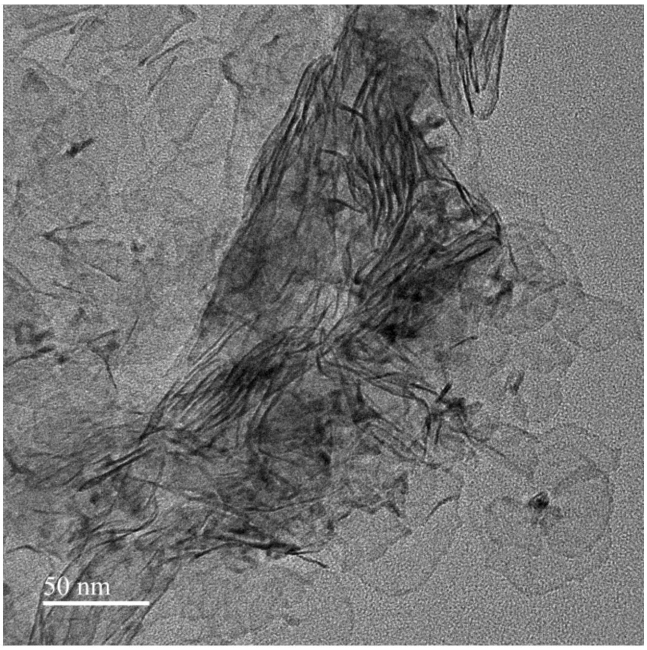

Figure S1 TEM image of PdMoCu metallene.

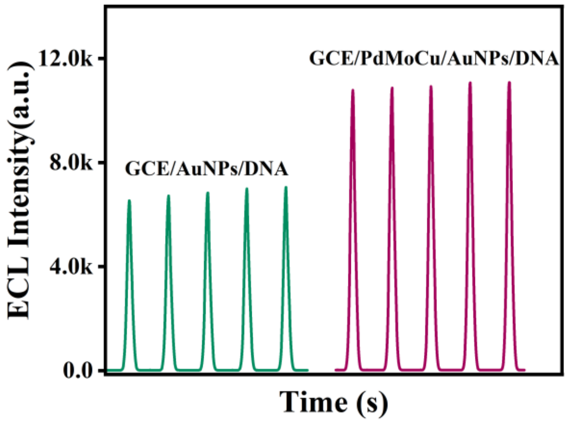

FigureS2 ECL Responses of Sensing Interfaces with and without PdMoCu Metallenes.

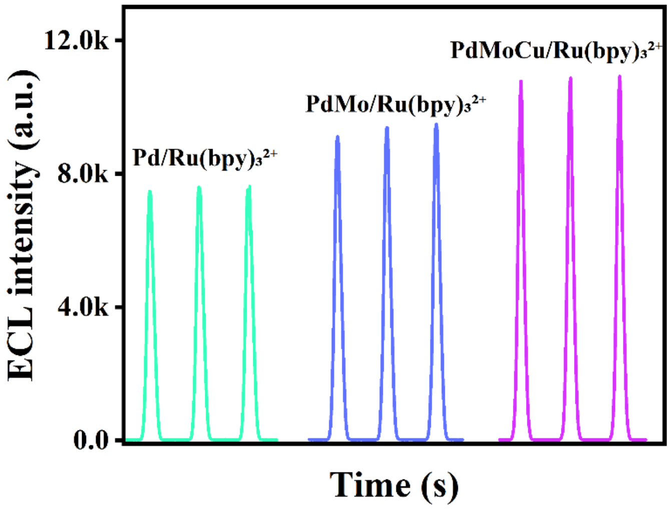

Figure S3 Comparison of ECL performance among monometallic (Pd), bimetallic (PdMo), and trimetallic (PdMoCu) systems.

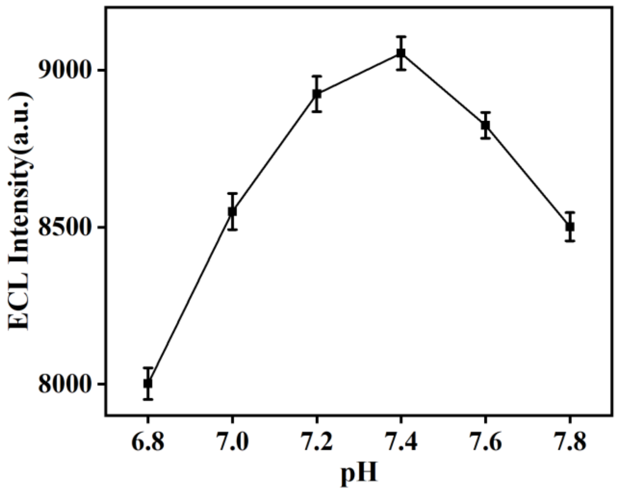

Figure S4. Optimization of experimental PBS pH affecting the ECL response.

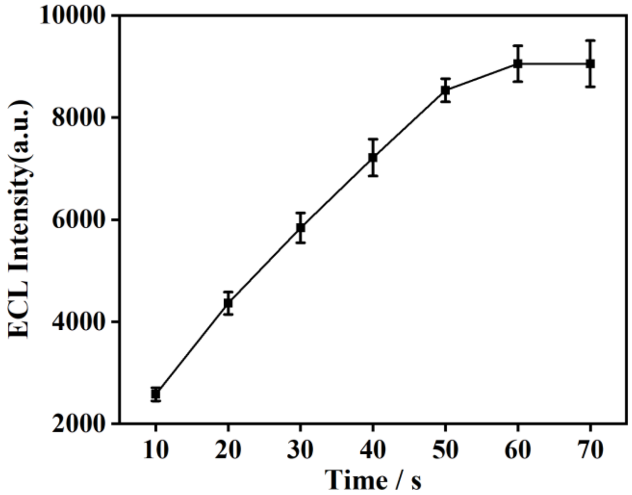

Figure S5. Effect of $Ru(bpy)_3^{2+}$ adsorption time on the ECL response of the DNA-modified electrode.

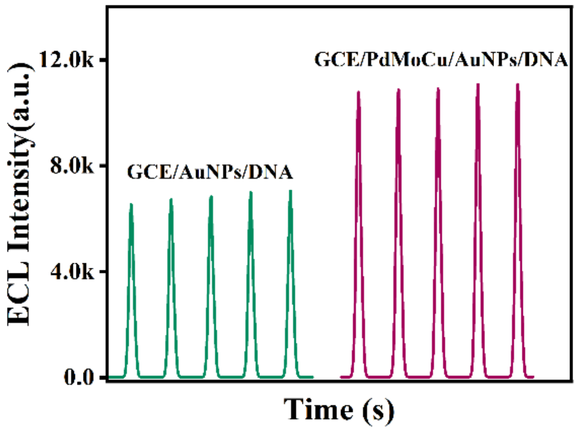

Figure S6 ECL signal near the LOD.

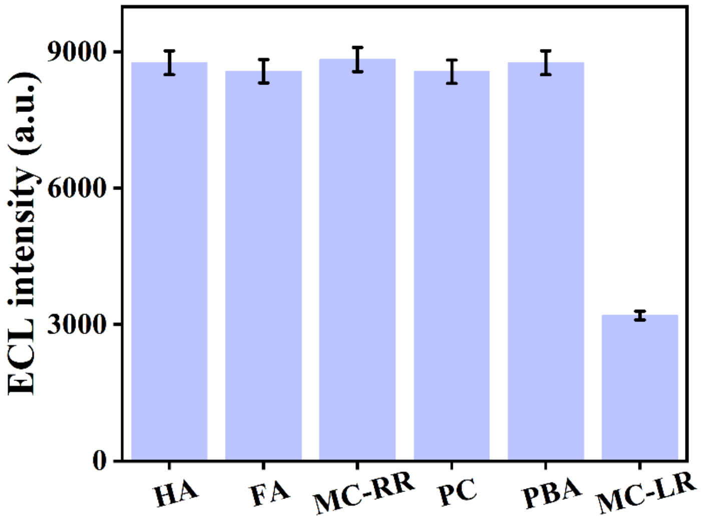

Figure S7 Selectivity evaluation of the ECL sensing platform

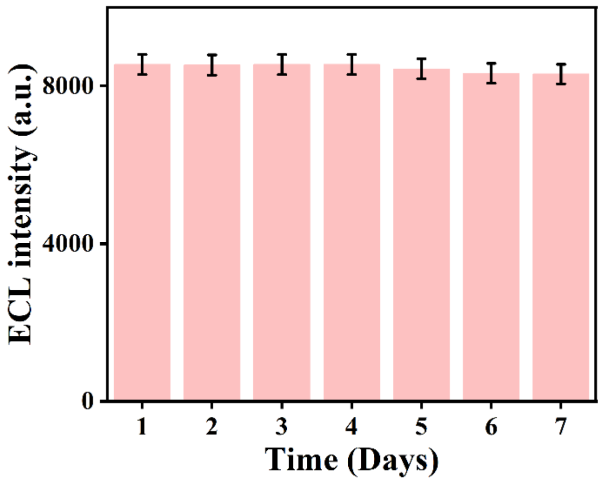

Figure S8 Stability test of the biosensor over 7 days at 4 °C

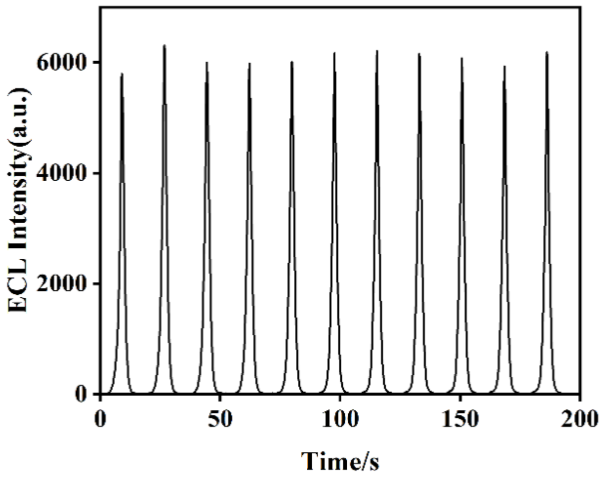

Figure S9. The stability test of the proposed ECL sensor.

Table S1 Quantitative characterization.

Step	R(Ω)	Rct(Ω)	Q(μF · sn−1)	n
(a)Bare GCE	50	100	22	0.88
(b) PdMoCu	52	1200	65	0.91
(c)PdMoCu/H1	55	2100	38	0.89
(d)PdMoCu/H1/Apt	58	3500	21	0.87
(e)+ $Ru(bpy)_3^{2+}$	56	2500	32	0.9
(f)MC-LR	59	2900	28	0.86

Table S2. Comparison of the analytical performance of different sensors for MC-LR detection.

Method	Linear range	LOD	Assay time	Complexity	Reference
DCC	0.25–10.00 ng/mL	0.08 ng/mL	84 min	laborious	36
CL	0.5–100.0 ng/mL	0.01 ng/mL	25 min	intricate	37
HPLC-MS	2.0–800.0 pg/mL	0.8 pg/mL	90 min	time-consuming	38
NLISA	1.0-500 μg/L	0.12 μg/L	45 min	laborious	39
ECL(RuCu)	0.0001–50 ng mL^{-1}	100 fg/mL	90 min	complicated	40
ECL (AuNPs)	0.1 pM – 1 nM	63.2 fM	130 min	lengthy	41
ECL	0.1 pg/mL-50 ng/mL	37 fg/mL	25 min	streamlined	This work

References

30. Feng, A.; Du, Z.; Zhou, Y.; Hua, Z.; Sun, Y.; Zhang, K. DNA Hydrogel-Templated Synthesis of Electrochemiluminescence Active Gold Nanoclusters with T7 Exonuclease Amplification for Ultrasensitive Microcystin-LR Detection. *Anal. Chem.* **2026**, *98*, 1628–1635.
31. Zhao, G.; Du, Y.; Zhang, N.; Li, Y.; Bai, G.; Ma, H.; Wu, D.; Cao, W.; Wei, Q. Bimetallic Metal-Organic Frameworks as an Efficient Capture Probe in Signal On-Off-On Electrochemiluminescence Aptasensor for Microcystin-LR Detection. *Anal. Chem.* **2023**, *95*, 8487–8495.
36. Peng Z, Kang X, Fan Z, et al. Capture-SELEX-based screening and mechanism analysis for the dual-colorimetric aptasening of microcystin in water [J]. *Analytica Chimica Acta*, **2025**, 1366: 344261.
37. Peng Z, Kang X, Fan Z, et al. Aptamer cleavage optimization, binding mechanism analysis, and dual-chemiluminescence sensor for microcystin detection. *Talanta*, **2025**, 292: 127948.
38. Zhang W, Wang Z, Zhang L. Interface-assisted synthesized covalent organic framework film for efficient extraction of microcystins in aquatic organisms. *Talanta*, **2025**, 282: 127051.
39. Zeng W, Lu M, Wu L, et al. Nanozyme mediated Raman-NLISA dual-modal immunosensor for accurate and sensitive detection of microcystin-LR. *Food Chemistry*, **2025**, 485: 144480.
